# Does less working time improve life satisfaction? Evidence from European Social Survey

**DOI:** 10.1186/s13561-022-00396-6

**Published:** 2022-09-30

**Authors:** Qinglong Shao

**Affiliations:** Institute of Chinese Studies, Freie Universität Berlin, Fabeckstr, 23-25, 14195 Berlin, Germany

**Keywords:** Life satisfaction, Working time, Ordered probit model, Health, Job category

## Abstract

**Background:**

Worktime is one of the main drivers of life satisfaction, and a balanced distribution of working hours and leisure hours directly impacts feelings of well-being. Based on previous studies, we seek to confirm this relationship in the European context and explore other potential driving forces of life satisfaction. Health condition as the mediating variable is also examined.

**Methods:**

This article uses an ordered probit model to analyze the impact of working time on life satisfaction using data extracted from the most recent round (wave 10) of the European Social Survey (ESS). Hypotheses are proposed to test the impact of working time on life satisfaction, the mediating effect of health in the worktime–satisfaction nexus, and the effects of social inclusion, social trust, feelings of safety, and digitalization on life satisfaction.

**Results:**

The results reveal a negative and significant correlation between hours of work and life satisfaction, thus implying that a shorter working week can improve Europeans’ life satisfaction. Health is found to be an important intermediate variable that plays an essential role in the dynamic through which working times influence life satisfaction. Further, we find that those in the middle class prefer to work shorter hours to achieve a higher feeling of satisfaction and that high earners to a lesser extent, while low earners generally show no preference. Employees of private firms are more satisfied with shorter working hours, while satisfaction for those working in public institutions is not affected by changes in hours worked. Finally, we verify the robustness of our estimations by replacing life satisfaction with happiness.

**Conclusions:**

Working fewer hours contributes to higher life satisfaction in Europe, and health plays an essential mediating role in this relationship. Social inclusion, social trust, feelings of safety and digitalization all play a factor in improving life satisfaction. Compared to other job categories, private sector employees can achieve greater life satisfaction from reducing their total working time.

## Highlights

➢ Ordered probit model is used to analyze worktime-satisfaction nexus in Europe.

➢ A shorter working schedule can improve life satisfaction.

➢ Health plays a mediating role in worktime-satisfaction nexus.

➢ Trust, social inclusion, safety and digitalization can promote life satisfaction.

➢ Private firm employees prefer shorter work weeks while others show no preference.

## Background

There is a small number of studies that theoretically explain and empirically analyze the determinants of life satisfaction [1, 2], and income has been identified as an important driver of satisfaction in numerous other studies [3, 4]. However, life satisfaction may also remain constant over time despite rising wealth [5, 6]. Therefore, “we must be highly skeptical of the view that long-term changes in the rate of growth of welfare can be gauged even roughly from changes in the rate of growth of output” [7]. On the contrary, economic recessions, for example, are likely to reduce psychological well–being, which entail not only declining income and increasing unemployment but also a sense of emotional loss [8]. Stress caused by the COVID-19 pandemic has been found to be strongly correlated with life satisfaction in Poland [9], and the mental health of jobless people in China should be of particular concern [10]. Personal characteristics, such as age, gender, and marital status, are also important influencing factors [11, 12]. The introduction of the European Working Time Directive (EWTD) has greatly reduced training hours for workers such as surgical residents, which has enhanced their job satisfaction [13].

In recent years, a growing number of empirical studies have explored the role of working time in well–being. Their findings are mixed, particularly on whether a shorter working week has positive or negative effects on well-being, and the call for more in-depth research remains unanswered. Using different methods, particularly the ordered probit and logit models, scholars have investigated the worktime–satisfaction nexus based on various national- and regional-level surveys conducted in, for example, the US, UK, Germany, Australia, France, Korea, and the EU [14]. We review the literature below to explore the nexuses between working time and work satisfaction and job satisfaction and overall life satisfaction. It is worth noting that life satisfaction is not necessarily correlated with job satisfaction [15]. The empirical literature on the worktime–satisfaction nexus is presented chronologically in Table 1.

**Table 1:** Summary of the literature on the worktime–satisfaction nexus (in chronological order)

Author(s)	Outcome variable(s)	Methods	Data structure	Main results
Weston et al. (2004) [16]	Life satisfaction; job satisfaction	Statistical correlation analysis	2001; Household, Income and Labour Dynamics in Australia (HILDA) Survey	Fathers working 35–40 h per week have the highest proportion of satisfaction, and the number of fathers who prefer to work fewer hours increases along with an increase in working hours. Fathers working more than 60 h who report high satisfaction have higher levels of well-being compared to those who are satisfied with a 35–40-h work week
Golden and Wiens-tuers (2006) [17]	Happy; satisfaction	Ordered logistic model	2002; General Social Survey (GSS) Quality of Working Life (QWL) module in the US	Monetary rewards for overtime work bring better mental health but no apparent increase in happiness. Work–family imbalances occur due to interference in workers' personal lives, but it is unclear whether happiness rises or declines when overtime work is mandatory
Clark and Senik (2006) [18]	Job satisfaction	Multivariate analysis; Ordered probit model	1991–2001; British Household Panel Survey (BHPS) 1994–2001; French component of the European Community Household Panel (ECHP)	Working hours and job satisfaction show opposite relations in the UK and France: a negative correlation (at 5% significance) occurs in the former, while a positive correlation (at 10% significance) is observed in the latter. This suggests that British people prefer a shorter working week, whereas long working time gives French people a sense of accomplishment
Pouwels et al. (2008) [19]	Happiness	Ordered probit model	1999; German Socio-Economic Panel (GSOEP)	The effect of income on happiness tends to be underestimated by 12% for women and 25% for men. Controlling for working hours would substantially increase the impact of income on subjective well-being
Booth and Ours (2008) [20]	Working hours satisfaction, job satisfaction, and life satisfaction	Fixed effect ordered logit model	1996–2003; BHPS	A standard full-time job (without overtime) can increase British men’s work satisfaction, but has no impact on their job satisfaction or life satisfaction. British women prefer part-time jobs, but their life satisfaction is unaffected by working hours
Booth and Ours (2009) [21]	Working hours satisfaction, job satisfaction, and life satisfaction	Fixed-effects ordered logit model	2001–2004; HILDA Survey	Women are happier with part-time jobs, and their partners working full-time can enhance their satisfaction. By comparison, the working hours of their partners show no significant impacts on men’s satisfaction, but working full-time themselves increases their life satisfaction by raising their prospects of success
Knabe and Rätzel (2010) [3]	Life satisfaction	Pooled ordered probit model; probit-adjusted OLS	1999–2006; GSOEP	For both men and women, the relationship between income and happiness is unaffected by including the working time variable because the impact of working hours on happiness is small. This finding differs from that of Pouwels et al. (2008)
Okulicz-Kozaryn (2011) [22]	Happiness	Pooled data ordered logistic model	1996, 2001; Eurobarometer survey series in Europe 1996, 1998, 2000, and 2002; GSS	Compared to Americans, Europeans are happier with less work. Both populations rationally seek to maximize their utility: Americans care more about work outcomes, while Europeans care more about work processes
Holly and Mohnen (2012) [23]	Life satisfaction, job satisfaction	Fixed effect regression; OLS	1999–2009; GSOEP	They find a positive relationship between life satisfaction and long working hours, while the desire to reduce working hours has a negative impact on satisfaction
Rudolf (2014) [1]	Subjective well-being	Fixed-effects ordered logit model	1998–2008; Korean Labor and Income Panel Study (KLIPS)	The evidence suggests that the Korean Five-Day Working Reform (i.e., reducing working hours) does not fulfil the expected aim of enhancing workers’ well-being. So a shorter working week does not necessarily make Koreans happier. Furthermore, rising work intensity may cancel out the increase in well-being
Collewet and Loog (2015) [24]	Life satisfaction	OLS and 2SLS with fixed effects	1985–2009; GSOEP	An inverted U-shaped effect of working hours on life satisfaction is found. However, the effect of full-time work on actual working hours for part-timers is too weak to consolidate. For full-time workers, increasing working hours may reduce life satisfaction in men but has no such impact in women
Valente and Berry (2016) [25]	Life satisfaction	Ordered logistic model	2008; Americas Barometer for Latin America; 2006, 2008, and 2010; GSS	Among overtime workers, married Latin American males are less happy than married US American males. This is explained based on social development theory: more work means improved welfare and higher status for US American men, whereas Latin American men are happier to enjoy family relationships
Wu (2016) [26]	Job satisfaction	Hierarchical regression analysis	2014–2015; questionnaire survey in Guangdong, Zhejiang, Shandong, and Jiangsu provinces, comprising 1,369 effective questionnaires	A U-shaped relation between working time and job satisfaction is found for three occupations in China: farmers, industrial workers, and public servants. On the one hand, workers’ health processes differ for equivalent hours of working. On the other hand, despite highly similar efforts, they acquire different incomes, which create an effort-income imbalance
Okulicz-Kozaryn and Golden (2017) [27]	Happiness	OLS	1998, 2002, 2006, 2010, and 2014; GSS pooled datasets	A flexible working schedule can substantially increase happiness; its effect can be compared to those of health and income, which are widely recognized as important drivers of happiness
Okulicz-Kozaryn and Golden (2018) [2]	Self-reported well-being	OLS	2016; GSS	For US citizens, the greater the instability and unpredictability of work schedules, the lower the workers’ subjective well-being is
Alameddine et al. (2018) [28]	Job satisfaction	Blinder-Oaxaca decomposition	1990–1995 and 1997–2015; The German Socio-Economic Panel	The mismatch between desired and actual working time negatively affects German nurses’ job satisfaction, and thus the authors propose bridging the gap between actual and desired work hours
Noda (2020) [29]	Life satisfaction	OLS	2014; the OECD Better Life Index	Leisure hours could improve Europeans’ life satisfaction, and this relationship is especially significant for men. The positive effect of health on life satisfaction is confirmed
Henriques et al. (2020) [30]	Life satisfaction	OLS	2011, 2012; the 3rd European Quality of Life Survey (EQLS)	Fewer working hours contribute to a higher level of life satisfaction in Europe, even to the point of sacrificing earnings, especially for workers with children
Tan et al. (2022) [31]	Work time satisfaction	Structural equation models	2012, ESS	Young children disrupt full-time working mothers’ but not full-time working fathers’ sleep. Compared to men, women report a significantly larger association between work hour dissatisfaction and restless sleep

### Working time and working hours satisfaction

Several scholars have investigated how worktime influences people’s satisfaction with their time spent at work from the perspective of gender. Booth and Ours [20] find that working full-time-and especially overtime-dissatisfies women, whereas men appear to have the highest working hours satisfaction if they work full-time, but not overtime. In a later study, the same authors consider interdependence within the family and focus on partnered men and women to investigate the cross-partner effects of part-time work on well-being. Their findings show that both women and men are more satisfied with their working hours if they work part-time [21]. To tackle the endogeneity problem, Rudolf [1] uses a fixed-effects ordered logit model to examine the worktime–satisfaction nexus. His results indicate that, for Korean wives, a shorter working week may raise their life satisfaction, which significantly declines if more working hours are required; likewise, overtime work can reduce the working hours satisfaction of Korean husbands. Moreover, women are likely to suffer disproportionately when both partners’ inter-role strain intensifies [31]. In sum, the empirical outcomes in various countries indicate that women have higher working hours satisfaction when working fewer hours, while men are satisfied with part-time or full-time jobs according to their own preferences. Both genders are clearly dissatisfied with overtime work.

### Working time and job satisfaction

In general, scholars have verified that a balanced worktime distribution between work and life increases satisfaction and health [32], and evidence shows that a mismatch between desired and actual working times negatively affects German nurses’ job satisfaction [28]. Regarding gender differences, Booth and Ours [20, 21] reveal a significant positive correlation between part-time work and job satisfaction in both British and Australian females, but not their male counterparts, thus implying that only women are generally happier when working fewer hours. Rudolf’s [1] findings in the Korean context confirm this relationship: job satisfaction significantly declines in wives required to work long hours, and overtime work can reduce husbands’ job satisfaction. He also tests cross-partner effects and finds that husbands working fewer hours can increase Korean wives’ job satisfaction. Using a German longitudinal dataset from 1999 to 2009, Holly and Mohnen [23] find a significant positive relationship between working hours and job satisfaction for all employees and separately for men and full-time workers. This suggests that employees, and particularly male employees, can achieve a feeling of accomplishment from their overwork. Therefore, it is not strange to observe a significant negative effect on job satisfaction only when employees want to reduce their working hours.

Wu [26] explores the relationship between working hours and job satisfaction based on the heterogeneity of efforts and rewards for three occupations in China: farmers, industrial workers, and public servants. He finds an inverted U-shaped relation between working hours and job satisfaction such that working moderate working hours (i.e., 6–7 h per day) maximizes job satisfaction, whereas longer or shorter working hours may reduce well-being. Since work contents and incomes vary across the three occupations, the impacts of working hours differ. Wu [26] finds that this relationship is stronger for farmers and public servants with high income than for industrial workers with high income. This may be attributable to factory employees being constantly engaged in repetitive physical work, such that their work and leisure are largely constrained by strict management regulations and overtime pay comes at the expense of their health. The situation differs for farmers and public servants. These observed differences in the interaction between occupations and the heterogeneity of working hours have important implications for China’s government, industries, and workers.

### Working time and overall life satisfaction

Researchers have examined the role of working time in the income–happiness nexus. Pouwels et al. [19] find that the wealth effect on happiness would be underestimated if the working time variable were to be excluded, and that this underestimation is significant for men but not for women. This suggests that worktime is important in determining happiness. Following that study, Knabe and Rätzel [3] re-examine their findings by expanding the 1999 German Socio-Economic Panel from cross-sectional data to a panel dataset with eight subsequent waves. Using the Probit-adjusted OLS, which is a more widely recognized method in the happiness literature, their results differ from those of Pouwels et al. [19] in that they find no supportive evidence that income’s impact on happiness tends to be downward biased without the worktime variable. Accordingly, they propose that working time only plays a peripheral role in determining happiness. In fact, it is common for research outcomes to be contradictory because researchers frequently use different methods and data.

Okulicz-Kozaryn [22] is the first to test empirically whether working less increases happiness more among Europeans than it does among Americans. The evidence confirms that this is the case, which he attributes to the fact that, in general terms, Americans care more about the work outcomes whereas Europeans place more value on work processes. This might be explained by the high competitiveness that characterizes the free market economy in the US. Valente and Berry [25] find that Latin Americans prefer part-time jobs, while US citizens prefer to work longer hours. This is compatible with the finding of Okulicz-Kozaryn [22] that US employees usually tend to work longer hours. Okulicz-Kozaryn and Golden [27] deepen their analysis by proposing that limited flexitime does not increase happiness and that a more flexible work schedule is needed to increase an individual’s life satisfaction. They also find in a later study in the US that the greater the instability and unpredictability of work schedules, the lower an individual’s subjective life satisfaction is [2]. An inverted U-shaped relation of working time and life satisfaction is found by Collewet and Loog [24], which implies that increasing working hours can enhance well-being, but beyond the peaking point of 37 h per week, well-being declines. However, the effects of working time on the life satisfaction of part-time employees are too weak to confirm. For full-time male workers, increasing working hours may reduce well-being, but this is not the case for full-time females.

Based on above discussions, it can be seen that these studies focus on the role of working time in the income–satisfaction nexus, and few studies have comprehensively explored the influence of working time on life satisfaction or sought to verify the mediating role of health and the effects of other essential driving forces such as social inclusion, social trust, feelings of safety, and digitalization. The worktime–satisfaction nexus in different job categories thus remains unexplored. To fill this gap, we use the recently released European Social Survey (ESS) data [33] to explore the correlation between working hours and life satisfaction among Europeans. In doing so, we make four contributions to the literature. First, we examine the promoting effect of working time on life satisfaction and the mediating effect of health in the worktime–satisfaction nexus. Second, the promoting effects of social inclusion, social trust, feelings of safety and digitalization on life satisfaction are examined. Third, the effect of wealth on working time is examined, and we show that income levels influence workers’ preferences with regard to working hours in Europe, with mid and high earners preferring to work less for a higher life satisfaction and low earners showing no preference. Fourth, the worktime–satisfaction nexus in multiple job categories is examined, while few studies focused on this point.

## Methods

### Hypotheses

#### The effect of working time on life satisfaction

The impact of working time on overall life satisfaction has been more extensively studied in the related literature. In advanced European countries, a work–life balance with enough leisure hours has been found to improve overall life satisfaction, and this relationship is especially significant among men [29, 30]. Other studies focus exclusively on overtime work. For instance, Holly and Mohnen [23] find that overtime work shows a highly significant positive effect on life satisfaction. Weston et al. [16] explore the impact of long working time on well-being for full-time employed fathers with partners and dependent children in Australia and find a negative correlation, with well-being declining as working hours increase. However, long working time is not necessarily associated with lower well-being for fathers working long hours because the extra income and feeling of accomplishment increase their happiness. Golden and Wiens-tuers [17] indicate that mandatory overtime work has mixed impacts on life satisfaction: being required to work extra hours increases satisfaction in some while reducing it in others. This effect depends on the interplay between the positive effects (e.g., worktime pay, sense of achievement, etc.) and negative effects (e.g., work-family interference, work stress, etc.). Clark and Senik [18] refer to the different structures of the French and British labour markets to explain the respective worktime–happiness nexuses in these two countries and find that the French are happier with more working hours, while the British prefer a shorter work week. Booth and Ours [20] examine the part-time work effect and find that women with children are happier if they can work part-time jobs for less than 15 h per week while raising children. They also find that men with children aged from 5 to 15 years are less happy than men with children of other ages. For couples without children, they find that part-time jobs make men happier, while the number of working hours has no impact on women’s life satisfaction. In this study, we use the actual working hours rather than contracted hours of work to explore their impact on European’s life satisfaction using the latest 2020 data.

- **H1**: Working time negatively affects life satisfaction.

#### The mediating effect of health on the worktime–satisfaction nexus

Although working time preferences differ substantially among individuals, overemployment (i.e., when actual hours exceed desired hours) has a significantly negative effect on workers’ health [28, 32]. Evidence indicates that longer working hours have an adverse impact on health [34], and work–life imbalance (i.e., a mismatch between desired and actual working hours) may also reduce employees’ self-perceived health conditions [35]. Noda [29] finds that self-reported health is a determinant factor that is positively associated with life satisfaction in OECD countries, and its impact on life satisfaction is not as significant as work–life balance due to the fact that Europeans take it good public health for granted. Thus, this relationship may be stronger than work–life balance in developing countries. In this study, health is employed as the intervening variable, and we examine its mediating role in the worktime–satisfaction nexus for Europeans using the latest ESS data.

- **H2**: Health is the mediating variable in the worktime–satisfaction nexus.

#### Other potential driving forces on life satisfaction

Recent studies focus on the impacts of certain personality traits on life satisfaction, such as self-reported social inclusion, social trust, feelings of safety and digitalization. People tend to experience high levels of life satisfaction when their physical, social, and psychological needs are met. Social inclusion, as a sense of being liked and accepted, is proven to be positively correlated with life satisfaction [36, 37]. Not surprisingly, home confinement during COVID-19 pandemic reduced people’s life satisfaction [38]. Social trust is positively associated with well-being, and it is a stronger determinant than income in advanced economies while this is not the case in developing ones [39]. In China, the happiness of males and urban residents is more likely to be affected by social trust than the happiness of female and rural residents [40].

Feelings of safety includes multiple aspects in terms of social, economic, and personal security. Most economic and social security depends upon familial solidarity and savings [41], but the welfare system also helps to provide this security [42]. Evidence in China confirms the effect of socio-economic security on life satisfaction [36]. Using the 2011 Swiss Crime Survey, Staubli et al. [43] confirm the detrimental effects of theft, attempted burglary and consumer fraud on happiness, and Kuroki [44] reveals that experiencing burglary and robbery reduced it in the Japanese context and that crime victimization hurts homeowners more than renters. In this study, we use European respondents’ data and expect to find a positive association between safety and life satisfaction. Digitalization may reduce social costs and enhance both work efficiency among government workers and convenience in people’s daily lives, thus promoting feelings of life satisfaction [45, 46]. Referring to Wang et al. [47], we use time spent on the internet to represent digitalization and investigate its impact on satisfaction. We expect to find a positive significant correlation, in line with recent studies.

- **H3**: Social inclusion is positively associated with life satisfaction.
- **H4**: Social trust is positively associated with life satisfaction.
- **H5**: Safety is positively associated with life satisfaction.
- **H6**: Digitalization is positively associated with life satisfaction.

#### Data collection and model specifications

The data are extracted from the ESS, which is an academically driven multi-country survey that has developed a series of social indicators, including attitudinal indicators. Ten ESS surveys have been conducted since 2002, and we use the latest (tenth) round survey in 2020 with 18,060 valid respondents. The life satisfaction question reads: *All things considered, how satisfied are you with your life as a whole nowadays?* We use this as the dependent variable in our analysis. The independent variables are the paid and unpaid working hours per week. Detailed survey questions and descriptions of the indicators are listed in Table 2. In addition to the life satisfaction and working time variables, we also include personal characteristic indicators, including health, social inclusion, social trust, feelings of safety, digitalization, income, marital status, gender, age, religion, and education. The mediating role of health is tested in this study and we further explore the worktime–satisfaction nexus in the three income levels (low-, mid- and high-income) and six job categories (central or local government, other public sector (such as education and health), state-owned enterprise, private firm, self-employed, and other). We divide income level into three equal groups with low-, mid- and high-income.

**Table 2:** Survey questions and descriptions of the variables from the ESS dataset

Variables	Survey questions	Responses
*Life satisfaction*	B27: All things considered, how satisfied are you with your life as a whole nowadays?	Scored from 0 to 10, where 0 means extremely dissatisfied and 10 means extremely satisfied
*Worktime*	F30: Regardless of your basic or contracted hours, how many hours do/did you normally work a week (in your main job), including any paid or unpaid overtime?	Hours worked per week, between 0 and 168 h
*Health*	C7: How is your health in general?	‘Very bad’ = 1; ‘Bad’ = 2; ‘Fair’ = 3; ‘Good’ = 4; ‘Very good’ = 5
*Social inclusion*	C4: Compared to other people of your age, how often would you say you take part in social activities?	‘Much less than most’ = 1; ‘Less than most’ = 2; ‘About the same’ = 3; ‘More than most’ = 4; ‘Much more than most’ = 5
*Digitalization*	A3: On a typical day, about how much time do you spend using the internet on a computer, tablet, smartphone or other device, whether for work or personal use?	Typical time spent on the internet per day, in minutes
*Trust*	A4: Using this card, generally speaking, would you say that most people can be trusted, or that you can’t be too careful in dealing with people?	Scored from 0 to 10, where 0 means you can’t be too careful and 10 means that most people can be trusted
*Safety*	C6: How safe do you—or would you—feel walking alone in this area after dark?	‘Very unsafe’ = 1; ‘Unsafe’ = 2; ‘Safe’ = 3; ‘Very safe’ = 4
*Income*	F41: Which letter describes your household’s total income, after tax and compulsory deductions, from all sources?	Scored from 0 to 10, where 0 means extremely low and 10 means extremely high, for weekly, monthly, and annual amounts
*Gender*	F2: Sex	‘Male’ = 1; ‘Female’ = 0
*Age*	F3: Age	Calculated by birth year
*Marital status*	F11: What is your legal marital status?	‘Yes’ = 1; ‘No’ = 0
*Religion*	C11: Do you consider yourself as belonging to any particular religion or denomination?	‘Yes’ = 1; ‘No’ = 0
*Education*	F15: What is the highest level of education you have successfully completed?	‘High school or lower’ = 1; ‘Bachelor’s degree’ = 2; ‘Master’s degree’ = 3; ‘Doctoral degree’ = 4
*Job category*	F32: Which of the types of organization on this card do/did you work for?	‘Central or local government’ = 1; ‘Other public sector (such as education and health)’ = 2; ‘A state-owned enterprise’ = 3; ‘A private firm’ = 4; ‘Self-employed’ = 5; ‘Other’ = 6

We recode *Education* as follows: we set 520 and below (including 520 and 000) to 1, between 610 and 620 to 2, between 710 and 720 to 3, and 800 and above to 4. *Marital status* is recoded, where 1 denotes married, otherwise it is 0. The *Health* variable includes both physical and mental health

Source: European Social Survey [33]

Robustness checks are applied by replacing life satisfaction with happiness such that the happiness question reads: *Taking all things together, how happy would you say you are?* Strictly speaking, life satisfaction and happiness have different connotations: the former reflects an individual’s cognitive judgment about the compatibility of living circumstances based on their own work and life experiences [48, 49], while the latter is a hedonic/emotional evaluation of their current state of mind [50]. For example, Lara et al. [51] regarded life satisfaction as the cognitive indicator of well-being and examined its association with current happiness. However, Schyns [52] found a close association between life satisfaction and happiness and suggests an interchangeable use of these two indexes. Mainstream literature follows this course and employs these two indexes to explain the individual’s subjective well-being [53–56]. Caner [57] estimates and compares the regression results using life satisfaction and happiness as outcome variables respectively. This study replaces life satisfaction with happiness to check its robustness. The reliability of our analysis is further verified if the outcomes after variable substitution are similar. Pairwise correlations for the dependent variables and the explanatory variables are reported in Table 3. The results illustrate two facts: first, most variables are significantly correlated at the 10% level, and second, working time is negatively correlated with life satisfaction as well as other explanatory variables except for gender and age. The observations highlight the importance of careful multivariate econometric analysis.

**Table 3:** Correlation analysis

Variables		1	2	3	4	5	6	7	8	9	10	11	12	13
*Life satisfaction*	**1**	1.0000												
*Worktime*	**2**	− 0.0238^a^	1.0000											
*Health*	**3**	0.3266^a^	0.0106	1.0000										
*Trust*	**4**	0.2728^a^	− 0.0632^a^	0.1330^a^	1.0000									
*Social inclusion*	**5**	0.1796^a^	− 0.0092	0.2313^a^	0.0792^a^	1.0000								
*Safety*	**6**	0.2172^a^	0.0016	0.1952^a^	0.1886^a^	0.0861^a^	1.0000							
*Digitalization*	**7**	0.0605^a^	0.0068	0.1074^a^	0.0187^a^	− 0.0024	0.0127	1.0000						
*Gender*	**8**	0.0004	0.0825^a^	0.0570^a^	− 0.0023	0.0376^a^	0.2135^a^	0.0079	1.0000					
*Age*	**9**	− 0.1338^a^	0.0240^a^	− 0.5000^a^	− 0.0388^a^	− 0.1175^a^	− 0.0843^a^	− 0.2988^a^	− 0.0539^a^	1.0000				
*Marital status*	**10**	0.0812^a^	0.0275^a^	− 0.0259^a^	0.0224^a^	0.0094	0.0637^a^	− 0.1376^a^	0.0352^a^	0.2053^a^	1.0000			
*Religion*	**11**	− 0.0686^a^	− 0.0013	− 0.1039^a^	− 0.1136^a^	0.0010	− 0.0556^a^	− 0.0849^a^	− 0.0746^a^	0.1652^a^	0.1213^a^	1.0000		
*Education*	**12**	0.1164^a^	− 0.0011	0.1286^a^	0.1104^a^	0.0916^a^	0.0401^a^	0.1666^a^	− 0.0369^a^	− 0.0495^a^	0.0600^a^	− 0.0089	1.0000	
*Income*	**13**	0.2714^a^	0.0396^a^	0.3336^a^	0.1708^a^	0.1460^a^	0.1575^a^	0.1435^a^	0.1120^a^	− 0.3415^a^	0.2665^a^	− 0.1128^a^	0.2882^a^	1.0000

^a^denotes that the correlation is significant at the 5% significance level (2-tailed). Job category is not included because it is not an ordinal variable and we use it to divide different groups

The ordered probit model was proposed by McElvey and Zavoina [58] for the analysis of categorical, non-quantitative choices, outcomes, and responses. To tackle the single crossing property problem inherent in standard logit/probit models (i.e., that the signs of the marginal effects can only change once when moving from the smallest to the largest categories), Boes and Winkelmann [59] propose four alternative models: the generalized threshold, random coefficients, finite mixture, and sequential models. The ordered probit model is suitable for this study [60] considering that the dependent variables—life satisfaction and happiness—are ordinal data that range from 0 to 10. More importantly, the ordered probit model takes into account unobserved heterogeneity and ordinarily in life satisfaction scales while using full information contained in the data [1]. As both the ordered probit and logit models are commonly employed to analyze such ordinal data, we choose the former since it is widely used in the related literature [18, 61, 62]. The basic equation of the ordered probit model is:

1 $${y}_{i}^{*}={X}_{i}{\beta }_{i }+ {\varepsilon }_{i}$$

where $y_{i}$ represents the dependent variable and ${y}_{i}^{*}$ the latent variable, denoting 11 levels of life satisfaction. ${X}_{i}$ is a vector of explanatory variables that assesses the attribution of life satisfaction, and ${\beta }_{i}$ is the coefficient of ${X}_{i}$, a vector of estimated parameters to be projected, which represents the impact magnitude of the independent on the dependent variables. Finally, ${\varepsilon }_{i}$ is unobserved white-noise disturbance, where $E\left({\varepsilon }_{i}\right)$.

Moreover, since the coefficients of the ordered probit model cannot be directly explained while the estimators are very similar to the ordinary least squares (OLS) model, we also construct the following alternative econometric specification following Ronning and Kukuk [63]:

2 $${Satisfaction}_{i}= {\alpha Worktime}_{i}+\Sigma {Individual}_{i}+ {\mu }_{i}$$

where ${Satisfaction}_{i}$ is the life satisfaction level reported by individual $i$, ${Worktime}_{i}$ is the reported working hours per week reported by individual $i$, $\Sigma {Individual}_{i}$ is the vector of the respondent’s individual characteristics, and ${\mu }_{i}$ is an error term. It is worth noting that the results are presented in forest plots to be visually friendly, referring to Becker and Kennedy [64], Lechner and Okasa [65] and Kostka et al. [66].

## Results

### The impact of working time on life satisfaction and the mediating effect of health

**Fig. 1:**
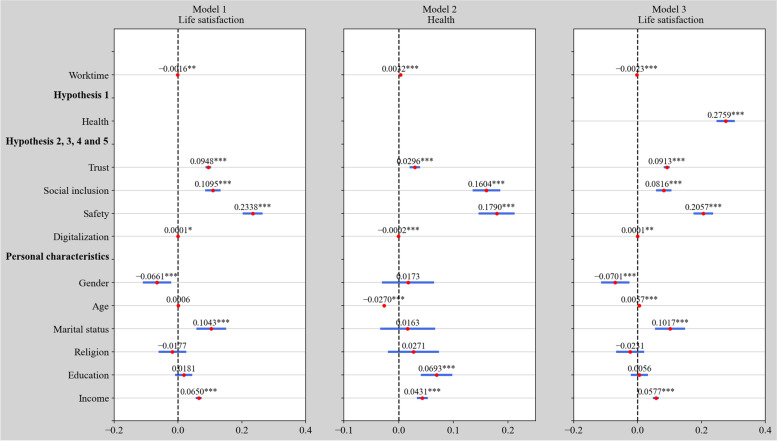
Estimation results of the impact of working time on life satisfaction and the mediating effect in 2020 using the ordered probit model. Notes: Red dots denote the coefficients; blue bars denote the 95% confidence interval; *, **, and *** denote p-values at 10%, 5%, and 1% significance levels, respectively. The same conventions are followed in all figures

To examine the relationship between weekly hours worked and self-reported life satisfaction, we present the estimation results of the ordered probit model in Fig. 1. All models control for a set of basic individual characteristics. In the basic estimation of Model 1, the weekly working time is negatively and significantly correlated with life satisfaction, thus implying that fewer working hours can raise life satisfaction. Two explanations can be offered for this finding. First, Europeans have a cultural norm of familyism and are happier working fewer hours to have more time to discharge family responsibilities and enjoy family relationships [25]. Second, the income tax rate is always high in European countries in order to support the welfare system. This suggests that “larger portions of labor earnings [are] being taken away, so the marginal return to labor [is] lower, disincentivizing European workers to labor longer” [67]. Thus, H1 is supported.

Model 2 and 3 test the mediating effect of health in the worktime–satisfaction nexus. In Model 2, working hours positively and significantly affect health, and health positively and significantly affects life satisfaction in Model 3. Thus, we identify a significant mediating effect of health in the worktime-satisfaction nexus referring to Wang et al. [47], which is also in line with Wu [26]. Among Americans, declining health is primarily responsible for driving down life satisfaction beyond midlife [68]. Therefore, H2 is confirmed. With regard to the four potential influencing factors, the results illustrate a positive significant impact of trust, social inclusion and feelings of safety on Europeans’ life satisfaction at the 1% significance level and the 10% significance level for digitalization, thus implying that a high-trust social environment, a life with numerous social activities, feelings of safety and more time spent on the Internet (include both the leisure and work hours) could improve life satisfaction. As such, H3-H6 are confirmed.

Regarding individual characteristics, income is found to be an important driver of life satisfaction at the 1% significance level. This effect has been confirmed by many prior studies [8, 19, 69, 70] and is generally interpreted as “more income brings greater happiness” [71]. Consistent with prior studies, age is positively and significantly correlated with life satisfaction in Model 3, which takes health into consideration, thus implying that elders are generally happier than those in their youth [19, 25]. This is influenced by the excellent social welfare system in Europe, as well as wealth accumulated over time. The negative effect of gender on life satisfaction implies that females are more likely to be happy than males and that marriage makes people happy. Religious people are not necessarily happier than non-religious people. Education shows no significant correlations with the dependent variable, which differs from the findings of Tella et al. [8] and reflects that the education–satisfaction nexus varies across countries. Possible explanations for this finding may be that people with higher levels of education are more likely to have higher salaries and more social status, but also have more responsibilities and heavier burdens, which may result in no net effect on life satisfaction.

### Worktime–satisfaction nexus in different income groups and job categories

**Fig. 2:**
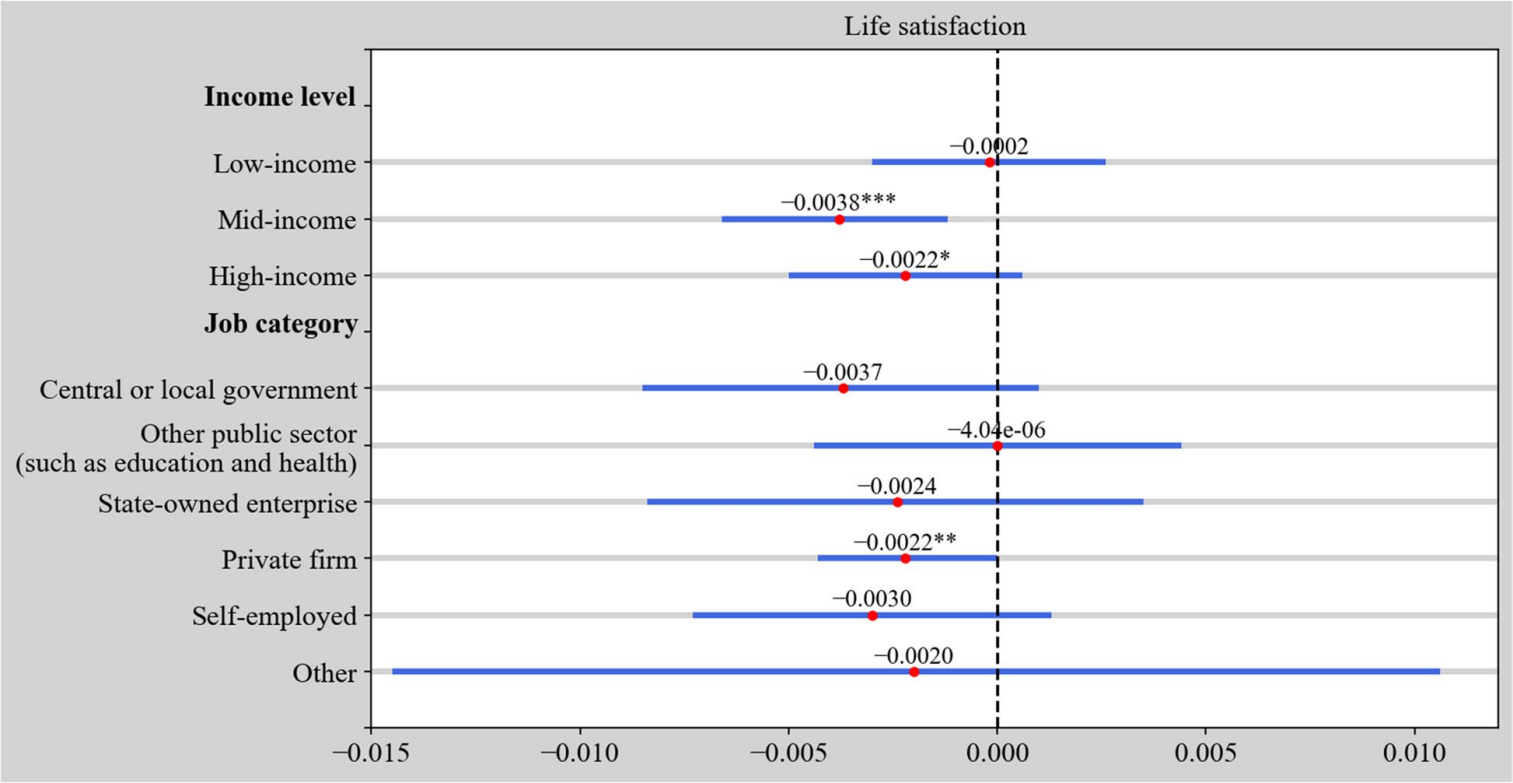
Estimation results of the impact of working time on life satisfaction at various income levels and job categories. Control variables are omitted

Prior research mainly focuses on the impact of income on life satisfaction, with very few studies analyzing how working time impacts satisfaction among different income groups and job categories. In this section, we aim to deepen our analysis by identifying the correlations between weekly working hours and self-reported life satisfaction for the three income groups and six job categories. The results are shown in Fig. 2. As can be seen, the worktime–satisfaction nexus is significant at the 1% level in the mid-income group and at the 10% level in the high-income group, which implies that mid and high earners tend to working less to achieve life satisfaction. This result is within our expectations because those in the middle-class must work more to accumulate more wealth whereas the marginal revenue of work is declining for those who already possess it. With regard to the various types of work, we find that employees of private firms prefer to work less to achieve a feeling of life satisfaction, while no significant relations are found in public institutions such as central/local government, education and health institutions or state-owned enterprises.

### Robustness check

**Fig. 3:**
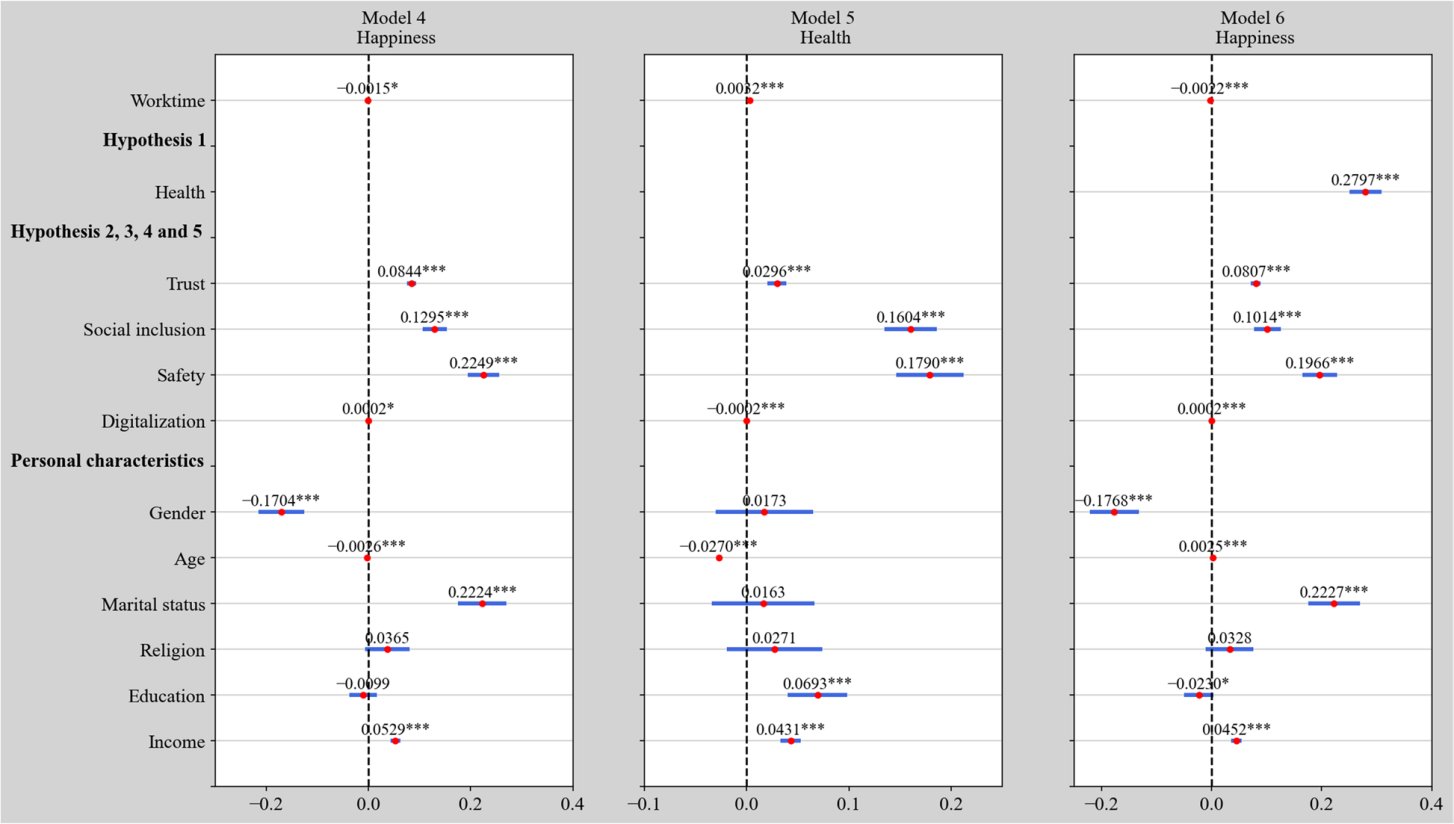
Estimation results of the impact of working time on life satisfaction and the mediating effect of health in 2020 using the ordered probit model

**Fig. 4:**
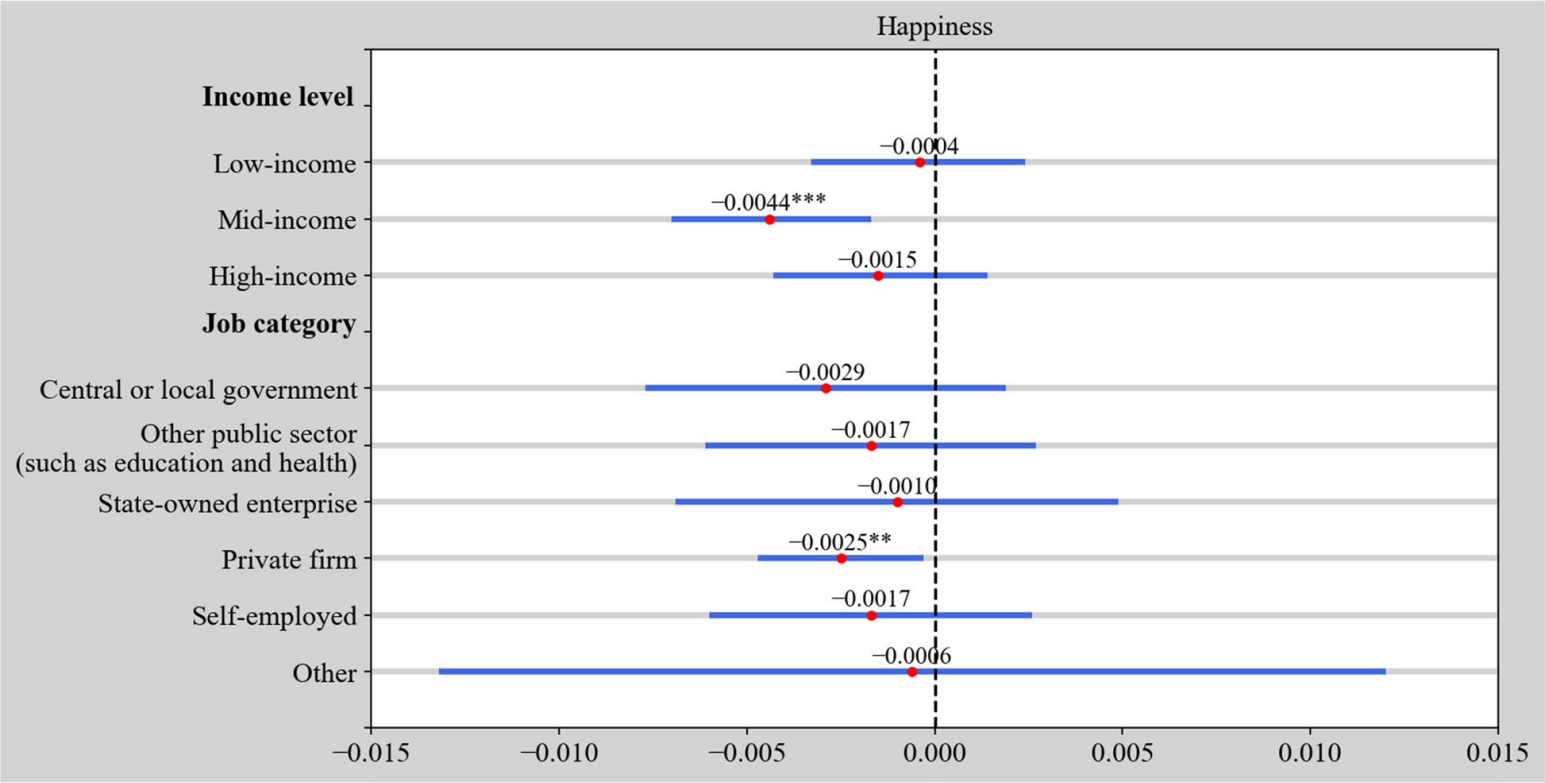
Estimation results of the impact of working time on happiness at various income levels and job categories. Control variables are omitted

This section checks the robustness of the above empirical results by replacing the life satisfaction variable with happiness. As shown in Figs. 3 and 4, the results are very similar to those in Figs. 1 and 2, thus confirming the robustness of our results.

## Discussion

Research on the determinants of life satisfaction have evolved from being income-driven to being driven by multiple factors that generally include those analyzed in this study (i.e., working hours, social trust, social inclusion, feelings of safety and digitalization). In advanced European countries, a balanced distribution of work and leisure hours is more important than income, as satisfaction comes from multiple economical, spiritual, and psychological sources, and economic satisfaction fulfils psychological needs by providing resources such as the leisure hours need to develop personal interests and care for the family. This likely explains why women prefer a shorter workweek, in that partnered women who work more hours still carry the burden of caring for the family, whereas very few men are primarily responsible for ordinary housework. Therefore, although women generally work as many total hours as men, they tend to prefer a shorter working week [72]. Moreover, if society cannot provide women with sufficient childcare and family-care hours or adequate pay, then it is not surprising to find increasing numbers of women working fewer hours to increase their well-being. Besides, an enhanced feeling of satisfaction is found among retired elderly people who do not have to work and can freely arrange their time, and their self-rated mental health increased as well [73].

Compared to Europeans, Americans’ satisfaction mainly derives from their work, particularly among mandatory rather than non-mandatory overtime workers, although both report higher stress than those who work no extra hours [17]. Rudolf [1] proposes that “workers with these very high hours are compensated with (non-observable) non-monetary rewards, such as higher status and decision-making power (wage-employed) or higher self-determination (self-employed).” Rothbard and Edwards [74] also point out, from a psychological perspective, that “instead of avoiding unpleasant role experiences, people actively try to solve the problems that make such experiences unpleasant, which requires investing time in those roles.” This suggests that the problem-solving effects are triggered by unpleasant experiences [75], and people prefer to tolerate working long hours in the short term not because they like those hours but because they anticipate increased utility in the long term. In this regard, long working hours, even when mandatory, can be seen as an investment in the US, which explains why mandatory overtime workers achieve more satisfaction than those working less.

Prior research has confirmed that self-rated health condition, including both mental [76] and physical health [77], is one of the main driving forces of life satisfaction (rather than the opposite, see Shields and Price [78]). For example, people with acute and chronic physical illnesses have lower levels of well-being [78], and disability can also reduce an individual’s life satisfaction [79]. In general, there are two possible ways in which physical health affects life satisfaction: the physical suffering caused by disease directly affects individual life satisfaction on one side; and on the other side, physical diseases cause psychological stress and affect satisfaction. Because of the worry and uncertainty about the disease, most patients will suffer from anxiety and depression; physical pain and psychological pressure interact with each other, forming a vicious cycle that jointly affects individual satisfaction. This effect is particularly prominent for the elderly, who are vulnerably affected by illness. Except for the high risks brought by physical diseases [80], self-rated life satisfaction is also confirmed to be significantly affected by mental health in the elderly population [81], and in certain conditions mental illness has a greater impact on satisfaction than physical illness [82]. Consider that approximately 15% of adults aged 60 and over suffer from a mental disorder, as reported by the World Health Organization [83], mental health problems in the elderly should not be ignored to guarantee the general life quality of the elderly. In light of this, actions should be taken to provide training for health professionals in providing care for older people and develop age-friendly services and settings. This also reminds the governments to put more emphasis on people’s mental health care in constructing the universal healthcare system and formulating the long-term healthy development plan.

Life satisfaction is also strongly affected by the frequency of engaging in social activities [84], and “the greater the extent of participation, the greater the degree of happiness reported” [85]. In fact, work is also a type of social participation. In a high-trust environment, individuals are generally convinced that the people around them, as well as the government, are honest. Such an environment can promote feelings of satisfaction. On the contrary, in a low-trust environment, people tend to worry more. They feel they must always be defensive in case others try to cheat, exploit or otherwise take advantage of them. This also relates to their feelings of safety, as happiness tends to be higher in areas with lower crime rates. In the current digital era, and especially during the COVID-19 pandemic, the impact of digitalization on life satisfaction is not as significant as the other three driving forces. This is because certain individuals may not achieve feelings of satisfaction by spending more time in internet, such as employees who work online. Thus, this factor exhibits a less significant correlation than the other three driving forces. Moreover, we observe that employees of private firms tend to prefer working less to achieve higher life satisfaction while changes in working time shows no impact for individuals who are self-employed and employees of public institutions. This is because approximately 81% of the respondents from private firms work 40 h or more, thus reducing their working hours could significantly improve their life satisfaction. In addition, approximately 65% of the employees of private firms are mid and high earners, thus overtime pay may not as important as leisure hours and they may find that a shorter workweek brings more satisfaction. This phenomenon may extend to countries outside of Europe because private firms often require their employees to work overtime, even without receiving additional compensation, while working time at public institutions is always fixed.

The existing literature suggests that people are generally dissatisfied by long working hours, particularly in advanced economies, and this study confirms this finding in the European context. Compared to developing countries, citizens of advanced countries have comparatively high earnings and are assisted by comprehensive welfare systems. Thus, the marginal returns to income are diminishing and a shorter workweek is likely to be more helpful in increasing their feelings of satisfaction. It is worth noting that this does not imply that income does not play a role in promoting life satisfaction; in fact, it shows a very strong effect for those in the middle-class because they require additional income to be upwardly mobile. The income effect in the rich group is not as strong as that in middle-class because their marginal returns to income are obviously diminishing considering their already-high incomes. Low earners in Europe always lose the motivation to work when they are well-cared for by the welfare system or lack professional skills.

## Conclusions

This study investigates the effect of working hours, as well as that of other driving forces, on life satisfaction using an ordered probit model based on the latest ESS data. The results show that working time is negatively associated with life satisfaction, which implies that Europeans generally prefer a shorter working week. Health plays an important role in the worktime–satisfaction nexus. Social trust, social inclusion, feelings of safety and digitalization show positive and significant effects on life satisfaction. In terms of income levels, mid and high earners prefer to work shorter work weeks while low earners show no preference. Employees of private firms prefer shorter work weeks while others show no preference. These findings complement the conventional views on working time and life satisfaction.

Several policies can be proposed based on the findings of this study. First, regulations that limit hours worked and protect employees’ health should be enacted or strengthened. Since health is an important factor in the relationship between working hours and life satisfaction, good physical and mental health can significantly improve life satisfaction. However, working either excessively long or too few hours may detrimentally affect life satisfaction; in the latter case, environmental pressures might be aggravated by a shorter working week [67]. Thus, it is important to restrict working hours to moderate levels in order to satisfy workers.

Second, economic development should be further promoted to build a digitalized society with low crime rates as well as high trust and social cohesion, especially under the current COVID-19 pandemic era. As the results show, these factors are significant driving forces on life satisfaction, while economic development is one of the main promoting forces of these factors, thus growth of economy is the key to improve the whole satisfaction level (this can be illustrated in the significant association between income and satisfaction). In specific, economic growth lowers crime rates partly due to increased employment [86]; social trust is able to affect long-term growth [87]; and social cohesion positively affects growth in multiple countries [88, 89]. Moreover, the pandemic lockdowns and social distancing measures greatly promoted online consumption and teleworking from home through virtual spaces, which objectively boosted digitalization [90, 91]. As such, digitalization is not a choice but a necessity.

Third, a strict implementation of new working time policies for private firms is needed. Typically, it is difficult to control overtime work in private firms, thus a targeted law is needed in this regard. Moreover, we should stimulate willingness to work and enhance life satisfaction-especially among low earners-it is necessary to increase employees’ overtime compensation. Evidence suggests that Chinese industrial workers are willing to work longer hours for a higher hourly income [26]. Moreover, this policy could help to narrow the gap between the rich and the poor, thereby tackling social inequality.

This study lays the groundwork for at least three future research directions. First and foremost, more sophisticated techniques, such as the fixed effects model, can be employed to avoid the potential endogeneity problem generated from reverse causality [1]. Second, a panel threshold model can be used to determine the thresholds beyond which longer or shorter working hours may decrease life satisfaction [67]. Third, factors other than health potentially play essential roles in the process through which working time affects life satisfaction. For example, the social inclusion indicator in our study shows significant signs across all models. We therefore propose that a shorter working week frees workers to participate in social activities to enhance their life satisfaction. Finally, few studies have examined the worktime–satisfaction nexus in different job categories. Though we briefly examine this issue in this study, the underlying reasons and concrete explanations call for further investigation. 

## Data Availability

Data are available under reasonable request.
